# Pan-immune-inflammation value and prognostic nutritional index can predict the 2-year recurrence of triple-negative breast cancer patients after radical resection

**DOI:** 10.3389/fonc.2026.1739129

**Published:** 2026-02-03

**Authors:** Zhenhua Xia, Yuping Dai, Jun Zhang, Liguo Yang, Zhigang Yang

**Affiliations:** 1 Department of Surgery II, Shidong Hospital of Yangpu District in Shanghai, Shanghai, China; 2 Department of Pathology, Shidong Hospital of Yangpu District in Shanghai, Shanghai, China

**Keywords:** pan-immune-inflammatory value, prognostic nutritional index, radical surgery, recurrence, triple-negative breast cancer

## Abstract

**Objective:**

This study investigates the value of Pan-Immune-Inflammatory Value (PIV) and Prognostic Nutritional Index (PNI) in predicting the recurrence of triple-negative breast cancer (TNBC) patients within 2 years after radical surgery.

**Methods:**

This study retrospectively selected the medical records of 130 TNBC patients who underwent radical surgery at Shidong Hospital of Yangpu District in Shanghai from January 2020 to March 2023. The preoperative PIV and PNI values and clinical data of the patients were collected, and the patients were divided into a recurrence group (47 cases) and a non-recurrence group (83 cases) based on the follow-up results within 2 years after surgery. The optimal cutoff values of PIV and PNI were determined through receiver operating characteristic (ROC) curves, and their predictive values for recurrence within 2 years after surgery were analyzed. Binary logistic regression models were used to analyze the independent predictive effects of PIV and PNI on recurrence within 2 years after surgery, and nomogram models were constructed to evaluate their clinical application value.

**Results:**

Tumor diameter (OR: 1.754, 95% CI: 1.156–2.663), SII (OR: 1.560, 95% CI: 1.053–2.312), PIV (OR: 1.891, 95% CI: 1.217–2.938), and PNI (OR: 0.676, 95% CI: 0.473–0.966) are factors affecting recurrence within 2 years after surgery in TNBC patients. Multivariate Cox regression analysis showed that low PNI (HR = 2.25, 95% CI: 1.40–3.62, P = 0.001) and high PIV (HR = 1.83, 95% CI: 1.17–2.86, P = 0.008) are independent risk factors for tumor recurrence within 2 years after surgery in TNBC patients. Patients with high PIV and low PNI (n=28) had the poorest RFS, while those with low PIV and high PNI (n=35) had the best outcomes. The calibration curve of the nomogram model constructed based on PIV and PNI showed a high consistency between the predicted and actual values.

**Conclusion:**

PIV and PNI can serve as effective indicators for predicting recurrence within 2 years after radical surgery in TNBC patients, providing a more scientific reference for clinicians.

## 1 Introduction

Triple-negative breast cancer (TNBC) is a special and highly aggressive subtype of breast cancer, characterized by the absence of estrogen receptor (ER), progesterone receptor (PR), and human epidermal growth factor receptor 2 (HER2) (1). TNBC accounts for approximately 10% to 20% of all breast cancers, but it has a higher rate of recurrence and metastasis, and a poorer prognosis. Since TNBC is not sensitive to hormone therapy or targeted therapy, it primarily relies on chemotherapy, which has limited efficacy, resulting in a lower 5-year survival rate for patients (2). Consequently, discovering effective biomarkers to forecast postoperative recurrence in TNBC patients is vital for refining treatment approaches and boosting patient results. Currently, several established biomarkers have been identified for TNBC prognosis. BRCA1 and BRCA2 germline mutations, present in approximately 15-20% of TNBC patients, are associated with improved sensitivity to platinum-based chemotherapy and PARP inhibitors, but their prognostic value remains controversial (3). Programmed death-ligand 1 (PD-L1) expression, detected in 20-40% of TNBC cases, has emerged as a predictive biomarker for immunotherapy response (4). Despite these advances, TNBC continues to exhibit the poorest 5-year overall survival rate among all breast cancer subtypes at approximately 77%, compared to 93% for hormone receptor-positive/HER2-negative and 85% for HER2-positive breast cancers (5). This underscores the urgent need for additional accessible and cost-effective prognostic indicators that can complement existing biomarkers and guide clinical decision-making.

Recently, as research on the tumor microenvironment has advanced, the significance of inflammation and immune response in tumor initiation, progression, and spread has increasingly gained attention. The Pan-Immune-Inflammatory Value (PIV) and the Prognostic Nutritional Index (PNI), as comprehensive indicators reflecting the systemic inflammation and nutritional status of patients, have been widely applied in the prognostic evaluation of various malignant tumors (6). PIV is an index calculated based on the counts of neutrophils, platelets, monocytes, and lymphocytes in peripheral blood, which can reflect the immune-inflammatory status of the body (7). Through serum albumin and peripheral blood lymphocyte counts, PNI measures the nutritional and immune condition of patients (8).

Studies have shown that PIV and PNI are closely related to the prognosis of patients with various cancers, including colorectal cancer, gastric cancer, and lung cancer (9). Recent meta-analyses have further demonstrated the prognostic value of combined inflammatory and nutritional indices across solid tumors, and validated the utility of PIV-PNI combination models in colorectal cancer. PIV is an index calculated based on the counts of neutrophils, platelets, monocytes, and lymphocytes in peripheral blood, which can reflect the immune-inflammatory status of the body (10). Through serum albumin and peripheral blood lymphocyte counts, PNI measures the nutritional and immune condition of patients (11). In the field of breast cancer, preliminary studies have explored the impact of PIV and PNI on patient prognosis. Moon et al. demonstrated that elevated inflammatory markers at five years post-diagnosis predict late recurrence in breast cancer patients. Şahin et al. found that low PIV predicts better chemotherapy response and survival in breast cancer patients receiving neoadjuvant therapy (12), while Ligorio et al. reported PIV’s prognostic significance in HER2-positive advanced breast cancer (13). However, Provenzano et al. recently showed that high PIV is associated with poor outcomes in advanced TNBC treated with platinum-based chemotherapy (14), suggesting potential but understudied relevance for early-stage TNBC. Critically, the independent prognostic value of combined PIV and PNI specifically for predicting early recurrence (within 2 years) after radical surgery in TNBC patients remains largely unexplored. This represents a significant knowledge gap, as TNBC exhibits distinct inflammatory and immunological profiles compared to other breast cancer subtypes, necessitating subtype-specific biomarker validation.

Therefore, this study aims to investigate the value of combined PIV and PNI as prognostic indicators for predicting early recurrence within 2 years after radical surgery in TNBC patients. Through retrospective analysis of clinical data from TNBC patients, the predictive ability of PIV and PNI for recurrence within 2 years after surgery was assessed, and their correlation with clinical pathological characteristics was analyzed. We hypothesize that low PNI and high PIV will independently predict increased recurrence risk, and that a combined nomogram model will demonstrate superior predictive accuracy compared to individual indices, providing a practical tool for clinical decision-making.

## 2 Materials and methods

### 2.1 Patient enrollment

The study was retrospectively conducted in accordance with the principles of the Declaration of Helsinki. The ethical approval was obtained by the ethics committee of Shidong Hospital of Yangpu District in Shanghai (approval No.:2025-025-01). The procedures used in this study were performed in accordance with the ethical standards as laid down in the 1964 Declaration of Helsinki and its later amendments or comparable ethical standards. Written informed consent was also obtained by all participants. The study included the medical records of 130 TNBC patients who underwent radical surgery at Shidong Hospital of Yangpu District in Shanghai from January 2020 to March 2023. The diagnosis of TNBC was in accordance with the guidelines of the American Society of Clinical Oncology/College of American Pathologists (ASCO/CAP) (15).

Inclusion criteria: 1) All patients were female, aged >18 years, and had complete and detailed clinical data and follow-up information; 2) The patients were pathologically diagnosed with invasive breast cancer, and immunohistochemical tests showed that ER, PR, and HER2 were all negative; 3) The patients had not received neoadjuvant chemotherapy, endocrine therapy, or radiotherapy before surgery.

Exclusion criteria: 1) Patients with other malignancies or a history of malignancy; 2) Those who had neoadjuvant chemotherapy, radiotherapy, endocrine therapy, or targeted therapy administered before undergoing surgery; 3) Those with severe infections, immune system conditions, or blood disorders that might affect inflammatory markers; 4) Women who are pregnant or breastfeeding; 5) Patients with incomplete clinical or follow-up details; 6) Patients with severe cardiac, hepatic, or renal insufficiency that would preclude treatment; 7) Exclusion of confounding factors that might interfere with the accuracy of the study results.

### 2.2 Data collection

The research utilized a retrospective structured approach, gathering information from the hospital’s electronic medical records and follow-up database for all women diagnosed with TNBC from January 2020 to March 2023. The inclusion process initially screened cases using the ICD-10 code C50 and keywords in laboratory immunohistochemical reports, followed by a review of the original pathology slide reports to confirm that ER and PR immunohistochemical staining were <1% and HER2 had no membrane staining or a FISH ratio <2.0. For patients who had received neoadjuvant therapy, pre-treatment core needle biopsy results were further retrieved to ensure accurate baseline molecular classification. The clinical variables were integrated from multiple sources, including the hospital’s HIS, LIS, PACS, and follow-up systems. The data collected included: Demographic information: Age, menopausal status, BMI, comorbidities. Tumor baseline characteristics: Tumor diameter, TNM staging (AJCC 8th edition), histological grade (Elston Ellis’s grading), Ki-67 index, number of lymph node metastases. Treatment plan: Breast-conserving radical surgery, total mastectomy, adjuvant chemotherapy. Laboratory indicators: Complete blood count, serum albumin, prealbumin, neutrophil-to-lymphocyte ratio (NLR), platelet-to-lymphocyte ratio (PLR), systemic immune-inflammation index (SII), PIV, PNI within one week before surgery. The PIV was calculated using the following formula: neutrophil count × platelet count × monocyte count/lymphocyte count. The PNI was calculated as: serum albumin (g/L) + 5 × lymphocyte count (×10^9^/L). All blood cell counts were obtained from preoperative peripheral blood samples collected within one week prior to surgery. Outcome events: The follow-up start point was the date of pathological diagnosis, and the endpoint was the patient’s recurrence status recorded two years later, as well as overall survival (OS), disease-free survival (DFS), sites of distant metastasis, and causes of death. Missing follow-up data were supplemented through telephone calls or outpatient visits. After exporting all data, a structured form was created using Excel 2019, with patient names and hospital numbers concealed, retaining only the research data. Data quality control was ensured by a third researcher who randomly reviewed 10% of the cases for manual verification, with a logical error rate of <1%, ensuring data integrity and consistency.

### 2.3 Radical surgery for TNBC

All patients included in this study underwent radical surgery under general anesthesia. The patients were placed in the supine position, and the surgical field was routinely disinfected and draped. The surgeon made a horizontal or vertical elliptical incision on the affected breast, dissected the skin flaps superiorly and inferiorly to the fascia of the pectoralis major muscle, and resected the entire breast tissue end bloc along with Level I-II axillary lymph node dissection, taking care to preserve the thoracodorsal nerve and axillary vein. After confirming negative surgical margins through rapid frozen section during the surgery, the wound was irrigated with warm distilled water, and thorough hemostasis was achieved. Negative pressure drainage tubes were placed in the axilla and along the sternum, and the subcutaneous tissue and skin were closed layer by layer. The surgery was concluded with compression dressing.

### 2.4 Data analysis

The study employed a dual-software approach using SPSS 26.0 and R 4.2.0 for data analysis. Continuous variables were first tested for normality using the Shapiro-Wilk test. Normally distributed variables were expressed as (Mean ± SD) and compared using independent samples t-tests, while non-normally distributed variables were described using the median and interquartile range (M (P25, P75)) and compared using the Mann-Whitney U test. Categorical variables were presented as n (%) and compared using the chi-square test or Fisher’s exact test as appropriate. Variables with *P* < 0.05 in univariate analysis were included in the multivariate Logistic/Cox regression models, and the forward LR method was used for variable selection. The results were reported as OR hazard ratios (HR) with 95% confidence intervals (CI). The Kaplan-Meier method was used for plotting survival curves, and the Log-rank test was applied to assess differences. To correct for multiple comparisons, the false discovery rate (FDR) correction threshold was set at *P* < 0.05. The nomogram model was developed with the rms package, and its predictive accuracy was internally validated through 1000 bootstrap resamples to determine the C-index and calibration curves, guaranteeing consistent and dependable predictions. The tests were two-sided, and statistical significance was assigned to *P* values under 0.05.

## 3 Results

### 3.1 Comparison of baseline characteristics between the two groups

This study included a total of 130 TNBC patients who underwent radical surgery. During the 2-year follow-up period, 47 patients (36.2%) experienced disease recurrence, while 83 patients (63.8%) remained recurrence-free. The mean age was 53.8 ± 10.2 years in the recurrence group and 52.1 ± 9.7 years in the non-recurrence group. The recurrence group had significantly higher tumor diameter, TNM stage, Elston-Ellis’s grade, Ki-67 index, NLR, PLR, SII, PIV, and proportions of lung, brain, bone, and liver metastases and lymph node metastases compared to the non-recurrence group (P<0.05). The PNI and lymphocyte count were significantly lower in the recurrence group than in the non-recurrence group (P<0.05). There were no significant differences between the two groups in age, BMI, hypertension, diabetes, cardiovascular disease, number of vascular invasions, surgical method, neutrophils, monocytes, platelets, serum albumin, prealbumin, menopausal status, etc. (P>0.05) (**Table 1**).

**Table 1:** Comparison of baseline data of the two groups of patients.

Factors	Recurrence group(*n* = 47)	Non-recurrence group(*n* = 83)	*t/x^2^*	*P*
Age (years)	53.8 ± 10.2	52.1 ± 9.7	0.94	0.174
BMI (kg/m²)	24.3 ± 3.1	23.9 ± 2.8	0.75	0.227
Comorbidities (n)				
Hypertension	18(38.3)	25(30.1)	0.91	0.341
Diabetes	8(17.0)	10(12.0)	0.62	0.430
Cardiovascular disease	5(10.6)	7(8.4)	0.03	0.855
Tumor Diameter (cm)	5.4 ± 1.5	4.5 ± 1.1	3.92	<0.0001
TNM Stage (n)			8.48	0.004
I-II	20(42.6)	57(68.7)		
III-IV	27(57.4)	26(31.3)		
Elston-Ellis Grade (n)			7.40	0.007
I-II	19(40.4)	54(65.1)		
III	28(59.6)	29(34.9)		
Ki-67 Index	42.1 ± 15.3	28.7 ± 12.5	5.41	<0.0001
Number of Vascular Invasion	1.6 ± 0.9	1.4 ± 0.6	1.52	0.066
Number of Lymph Node Metastases	3.2 ± 1.1	3.1 ± 1.3	0.44	0.328
Surgical Method			0.21	0.644
Breast-conserving radical surgery (n)	14(29.8)	28(33.7)		
Total mastectomy (n)	33(70.2)	55(66.3)		
Neutrophils (×10^9^/L)	5.1 ± 1.4	4.9 ± 1.1	0.90	0.185
Lymphocytes (×10^9^/L)	1.3 ± 0.4	1.5 ± 0.5	-2.35	0.010
Monocytes (×10^9^/L)	0.52 ± 0.18	0.51 ± 0.15	0.34	0.367
Platelets (×10^9^/L)	241.1 ± 61.8	238.2 ± 52.4	0.28	0.388
Serum Albumin (g/L)	38.9 ± 4.2	39.2 ± 3.9	-0.41	0.341
Prealbumin (mg/L)	228.2 ± 45.6	235.9 ± 41.3	-0.98	0.164
NLR	4.1 ± 1.6	3.6 ± 1.1	2.10	0.019
PLR	156.2 ± 52.0	128.6 ± 38.1	3.47	0.0003
SII	505.4 ± 28.1	479.4 ± 25.3	5.41	<0.0001
PIV	385.7 ± 47.1	352.8 ± 48.3	3.76	<0.0001
PNI	45.2 ± 4.6	50.1 ± 4.3	-6.09	<0.0001
Menopausal Status (n)			0.35	0.554
Yes	28(59.6)	45(54.2)		
No	19(40.4)	38(45.8)		
Lung Metastasis (n)	11(23.4)	0	21.2	<0.0001
Brain Metastasis (n)	6(12.8)	0	11.1	0.001
Bone Metastasis (n)	8(17.0)	0	15.2	<0.0001
Liver Metastasis (n)	9(19.1)	0	16.8	<0.0001
Lymph node metastasis (n)	30(63.8)	20(24.1)	19.5	<0.0001

Data are presented as mean ± standard deviation, or number (percentage) as appropriate.

### 3.2 Logistic regression analysis of factors influencing recurrence in TNBC patients two years after surgery

A binary logistic regression analysis was performed with recurrence within two years after surgery (coded as 1 for recurrence and 0 for no recurrence) as the dependent variable. Variables from **Table 1** that were significant in the univariate analysis (with P ≤ 0.05 for inclusion and P>0.05 for exclusion) were included as independent variables. Continuous variables such as tumor diameter, Ki-67 index, NLR, PLR, SII, PIV, and PNI, as well as lymphocyte count, were entered as measured values. Metastasis indicators for lung, brain, bone, liver, and lymph nodes (coded as 1 for presence and 0 for absence) were also included. TNM stage (I-II=1, III-IV=0) and Elston-Ellis’s grade (I-II=1, III = 0) were coded accordingly. The logistic regression analysis showed that tumor diameter (OR: 1.754, 95% CI: 1.156–2.663), SII (OR: 1.560, 95% CI: 1.053–2.312), PIV (OR: 1.891, 95% CI: 1.217–2.938), and PNI (OR: 0.676, 95% CI: 0.473–0.966) were factors affecting recurrence within two years after surgery in TNBC patients (**Table 2**).

**Table 2:** Logistic regression analysis of factors influencing recurrence in TNBC patients two years after surgery.

Factors	*B*	*S.E.*	*Wald*	*P*	*OR*	95%*CI*
Tumor Diameter	0.562	0.213	6.947	0.008	1.754	1.156 – 2.663
Ki-67 Index	0.218	0.176	1.537	0.215	1.243	0.880 – 1.756
NLR	0.301	0.189	2.533	0.111	1.351	0.933 – 1.957
PLR	0.198	0.165	1.441	0.230	1.219	0.883 – 1.683
SII	0.445	0.201	4.902	0.027	1.560	1.053 – 2.312
PIV	0.637	0.225	8.021	0.005	1.891	1.217 – 2.938
PNI	-0.392	0.182	4.641	0.031	0.676	0.473 – 0.966
Lymphocyte count	-0.208	0.171	1.479	0.224	0.812	0.581 – 1.135
Lung Metastasis (n)	0.410	0.267	2.356	0.125	1.507	0.892 – 2.545
Brain Metastasis (n)	0.785	0.312	4.343	0.062	2.192	1.189 – 4.040
Bone Metastasis (n)	0.369	0.243	2.304	0.129	1.446	0.898 – 2.328
Liver Metastasis (n)	0.421	0.289	2.122	0.145	1.523	0.865 – 2.682
Lymph node metastasis (n)	0.512	0.213	3.776	0.116	1.669	1.099 – 2.535
TNM Stage	0.603	0.234	3.637	0.107	1.828	1.156 – 2.891
Elston-Ellis Grade	0.418	0.198	3.456	0.085	1.519	1.031 – 2.238

### 3.3 Predictive value of PNI and PIV for recurrence within two years after surgery in TNBC patients

**Figure 1:**
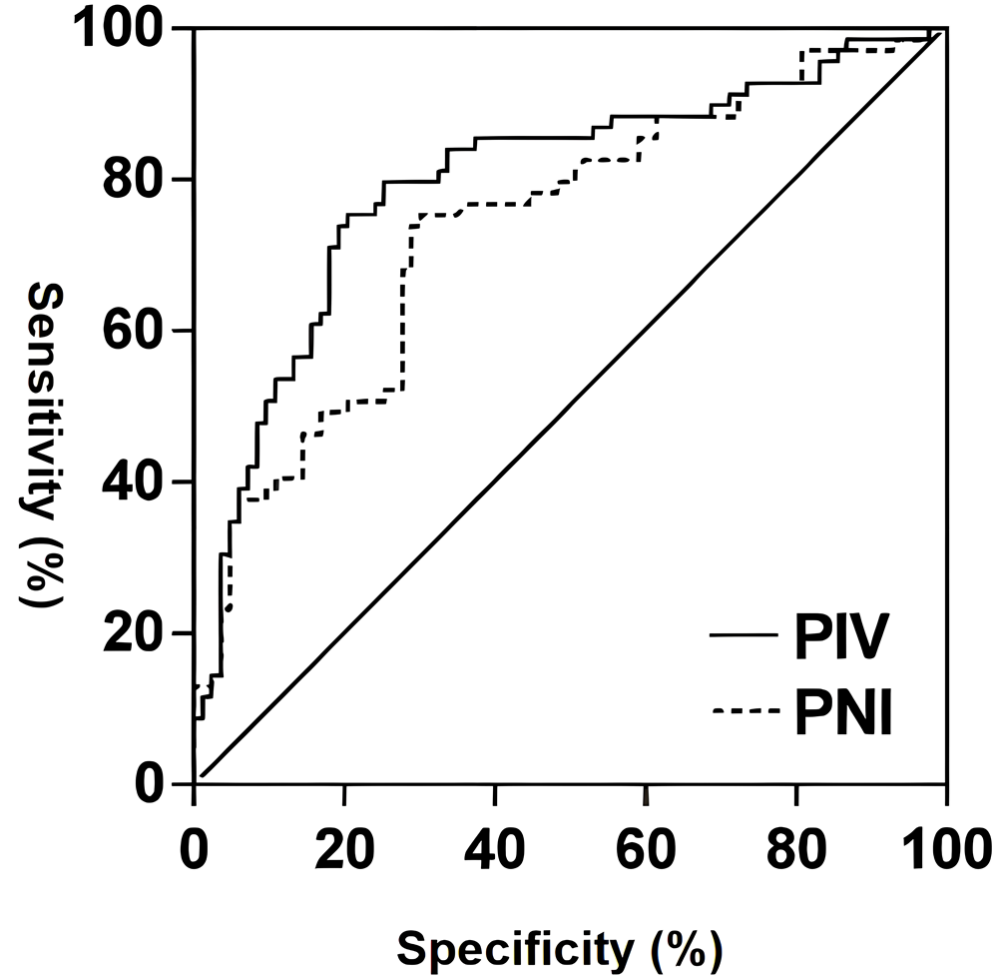
The ROC curves of PNI and PIV for the predictive value of recurrence in TNBC patients two years after surgery.

The corrected curve (AUC) for predicting recurrence within two years after surgery in TNBC patients was 0.762 for PNI. When the optimal cutoff value for PNI was set at 50.72, the specificity was 0.701 and the sensitivity was 0.762. The AUC for predicting recurrence within two years after surgery in TNBC patients was 0.799 for PIV. When the optimal cutoff value for PIV was set at 380, the specificity was 0.789 and the sensitivity was 0.760. Based on these cutoffs, patients were categorized into low PNI (<50.72) and high PNI (≥50.72) groups, and high PIV (≥380) and low PIV (<380) groups (**Figure 1**).

### 3.4 Cox regression analysis of recurrence of TNBC patients two years after surgery among different subgroups

Multivariate Cox regression analysis showed that low PNI (HR = 2.25, 95% CI: 1.40–3.62, *P* = 0.001) and high PIV (HR = 1.83, 95% CI: 1.17–2.86, *P* = 0.008) were independent risk factors for tumor recurrence within 2 years after surgery in TNBC patients, indicating that nutritional status and systemic inflammation levels have important value in predicting TNBC recurrence (**Table 3**).

**Table 3:** Cox regression analysis of recurrence in TNBC patients at two years after surgery among different subgroups.

Influencing factors	*B*	*S.E.*	*Wald*	*P*	*HR*	95%*CI*
Low PNI	0.812	0.241	11.34	0.001	2.252	1.403 – 3.615
High PNI	0.018	0.008	4.1	0.064	1.018	1.002 – 1.034
Low PIV	0.049	0.015	3.8	0.0097	1.05	1.02 – 1.08
High PIV	0.603	0.228	6.99	0.008	1.828	1.170 – 2.857

### 3.5 Nomogram prediction model for recurrence of TNBC patients two years after surgery

**Figure 2:**
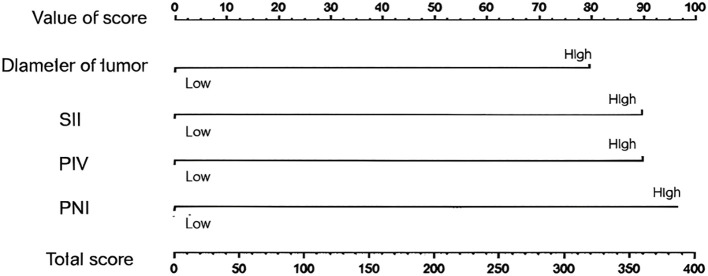
Nomogram prediction model for recurrence of TNBC patients two years after surgery.

**Figure 3:**
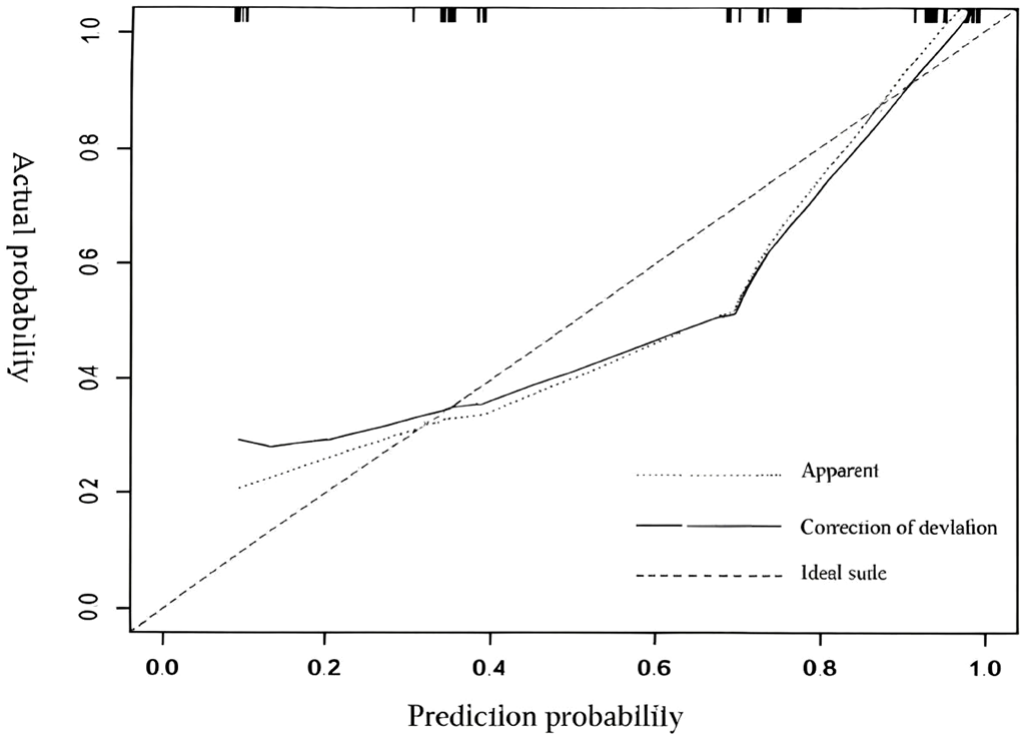
Calibration curve of the nomogram prediction model.

Based on the results of Logistic regression analysis, a nomogram prediction model for the recurrence of TNBC patients two years after surgery was constructed. The independent value of PNI and PIV in the prediction of TNBC recurrence, with HR values of 2.25 and 1.83 corresponding to score weights of 57.4 and 42.6 respectively, is consistent with the statistical significance results of 95%CI 1.40-3.62 and 1.17-2.86. (**Table 3**). The calibration curve shows that the predicted values of the nomogram prediction model have a high consistency with the actual values, indicating that the model has a high degree of accuracy (**Figures 2**, **3**).

### 3.6 Kaplan-Meier survival analysis

**Figure 4:**
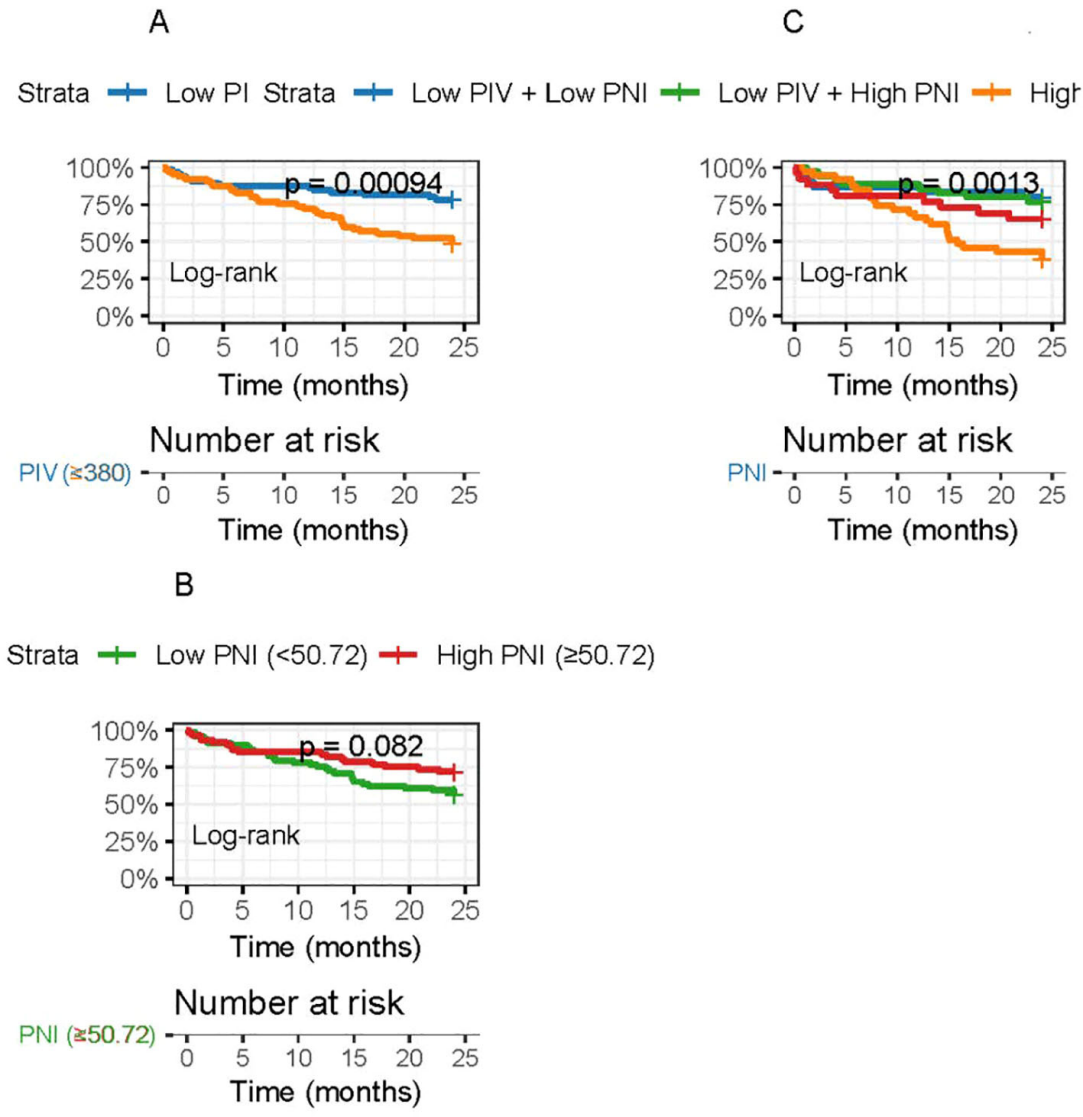
Kaplan-Meier curves for recurrence-free survival (RFS) in TNBC patients post radical surgery. **(A)** RFS comparison between low PIV (<380) and high PIV (≥380) groups. **(B)** RFS comparison between low PNI (<50.72) and high PNI (≥50.72) groups. **(C)** RFS comparison among four combined groups stratified by both PIV and PNI cutoffs. P values were calculated using the log-rank test.

Kaplan-Meier survival curves demonstrated significant differences in recurrence-free survival (RFS) between patient groups stratified by PIV and PNI cutoffs. The high PIV group (≥380) showed significantly shorter RFS compared to the low PIV group (<380) (log-rank test, P<0.001). Similarly, the low PNI group (<50.72) exhibited significantly worse RFS than the high PNI group (≥50.72) (log-rank test, P<0.001). When combining both indices, patients with high PIV and low PNI (n=28) had the poorest RFS, while those with low PIV and high PNI (n=35) had the best outcomes (log-rank test, P<0.001) (**Figure 4**).

## 4 Discussion

This research retrospectively examined the clinical data of 130 TNBC patients to assess how well PIV and PNI can predict recurrence within two years post-surgery and to explore their relationship with clinical pathological features. The results showed that tumor diameter, SII, PIV, and PNI are factors affecting recurrence within 2 years after surgery in TNBC patients. Multivariate Cox regression analysis revealed that low PNI and high PIV are independent risk factors for tumor recurrence within 2 years after surgery in TNBC patients.

In breast cancer, emerging evidence has identified the prognostic value of systemic inflammation and nutritional indices. Moon et al. demonstrated that elevated NLR at five years post-diagnosis predicts late recurrence in breast cancer patients, highlighting the long-term impact of chronic inflammation on disease course. Şahin et al. reported that low PIV predicts better chemotherapy response and survival in breast cancer patients receiving neoadjuvant therapy, though their cohort included mixed subtypes and advanced-stage disease. Ligorio et al. specifically validated PIV’s prognostic significance in HER2-positive advanced breast cancer, suggesting subtype-specific utility. Provenzano et al. recently established that high PIV correlates with poor outcomes in advanced TNBC treated with platinum-based chemotherapy. However, these studies predominantly focused on advanced or metastatic disease, heterogeneous breast cancer populations, or non-TNBC subtypes. Our study is among the first to evaluate the combined prognostic value of PIV and PNI specifically for predicting early recurrence after radical surgery in early-stage TNBC patients (16). By demonstrating independent associations and developing a validated nomogram, we provide the first tailored prognostic tool for this high-risk surgical population (17).

In this study, PIV and PNI demonstrated high accuracy in predicting recurrence within 2 years after surgery for TNBC patients. Moreover, low PNI and high PIV were identified as independent risk factors for tumor recurrence within 2 years after surgery in TNBC patients, indicating that nutritional status and systemic inflammation levels hold significant value in predicting TNBC recurrence. PNI and PIV have been widely applied in the prognostic evaluation of various cancers. Patients with hepatocellular carcinoma have shown that PNI is a key predictor of survival without disease and overall survival after undergoing surgery (18). In patients with soft tissue sarcoma and gastrointestinal stromal tumors, PNI has also shown its independent predictive value in forecasting long-term survival and recurrence risk (19, 20). These studies suggest that PNI holds significant prognostic value across different types of cancer, providing theoretical support for its application in TNBC. Patients suffering from nasopharyngeal carcinoma showed a significant association between PIV and decreased overall survival and progression-free survival (21). In colorectal cancer, PIV was correlated with the risk of distant metastasis in patients with KRAS mutations, but not in those with wild-type KRAS (22). These observations show that PIV may be a valuable resource for determining the prognosis of individuals with cancer. The successful application of PIV and PNI in various cancers provides strong support for their predictive value in TNBC. Combining various biomarkers with clinical characteristics to build a more detailed predictive model will aid in providing more accurate personalized treatment (23, 24).

Our nomogram, integrating PIV and PNI, provides a practical tool for preoperative risk stratification in TNBC patients undergoing radical surgery. By objectively quantifying systemic inflammatory and nutritional status, clinicians can identify the 36.2% of patients at high risk for early recurrence who may benefit from intensified adjuvant therapy or closer surveillance. Notably, the model’s high calibration accuracy suggests its utility in counseling patients about prognosis and tailoring follow-up schedules. Future prospective studies should evaluate whether nutritional support to improve PNI and anti-inflammatory interventions to reduce PIV can modify recurrence risk, potentially transforming these biomarkers from prognostic indicators to therapeutic targets. Multi-center validation and integration with tumor genomic profiling will further refine risk prediction and guide precision medicine approaches in TNBC.

The prognostic value of PIV and PNI in TNBC is biologically grounded in their reflection of the dynamic interplay between tumor cells and the host microenvironment. NBC is characterized by a uniquely aggressive tumor microenvironment marked by high proliferation rates, extensive necrosis, and dense immune infiltration, all of which generate substantial systemic inflammatory responses.

The high PIV observed in recurrent TNBC patients integrates neutrophil, platelet, and monocyte counts—key cellular mediators of cancer-associated inflammation. In TNBC, neutrophils release neutrophil extracellular traps (NETs) that promote metastatic seeding and chemoresistance through direct chromatin-mediated activation of cancer cells and shielding circulating tumor cells from immune surveillance. Platelets facilitate tumor cell epithelial-mesenchymal transition (EMT) and support the survival of circulating tumor cells in the bloodstream, a critical step in TNBC’s metachronous metastasis (25). Monocytes differentiate into tumor-associated macrophages (TAMs) that constitute up to 50% of the tumor stroma in TNBC, secreting pro-angiogenic factors like VEGF and immunosuppressive cytokines such as IL-10 and TGF-β. Our finding that high PIV independently predicts recurrence (HR = 1.83) suggests that this composite inflammatory burden outweighs the contribution of individual cell types, capturing the synergistic protumor genic ecosystem that defines aggressive TNBC.

PNI, combining albumin and lymphocyte counts, serves as a proxy for host immune-nutritional reserve. Hypoalbuminemia in cancer reflects not only malnutrition but also systemic inflammatory consumption and hepatic reprioritization of acute-phase protein synthesis (26). In TNBC, where cancer cachexia develops rapidly due to high metabolic demand, albumin levels mirror the host’s capacity to tolerate adjuvant chemotherapy and mount anti-tumor immunity. Lymphopenia, the second component of PNI, directly compromises cell-mediated cytotoxicity against TNBC cells. Our observation that low PNI more than doubles recurrence risk (HR = 2.25) underscores that immunocompromised and nutritionally depleted hosts cannot effectively eradicate minimal residual disease despite radical surgery.

The hypoxic and immunosuppressive TNBC microenvironment is further exacerbated by systemic inflammation and malnutrition. Hypoxia-inducible factors (HIFs) upregulated in TNBC induce PD-L1 expression, fostering immune evasion. Concurrently, inflammatory cells recruited by tumor-derived cytokines produce reactive oxygen species that promote genomic instability, while nutritional deficits impair DNA repair mechanisms (27). Thus, PIV and PNI likely capture a feed-forward loop where aggressive TNBC biology induces systemic inflammation and catabolism, which in turn compromises host anti-tumor surveillance, enabling early recurrence within the critical 2-year window. This mechanistic framework positions PIV and PNI not merely as prognostic markers but as potential therapeutic targets, nutritional intervention and anti-inflammatory strategies could improve outcomes in high-risk patients identified by our nomogram.

Investigations into PIV and PNI in colorectal cancer have demonstrated that these indicators correlate with the risk of distant metastasis in patients who have KRAS mutations, but not in those with the wild-type KRAS (28). This suggests that PIV and PNI may have different impacts on cancer progression under specific genetic backgrounds, and such genetic differences may also apply to TNBC. In TNBC, a hypoxic environment is considered one of the key factors leading to chemotherapy resistance and recurrence. Studies have shown that TNBC cells can enrich a cancer stem cell-like phenotype under hypoxic conditions, and this phenotype persists after reoxygenation, promoting resistance to chemotherapy and recurrence (29). The potential mechanisms of PIV and PNI are echoed here, as these indicators could reflect the immune and nutritional status within the tumor microenvironment, which may influence drug resistance and the tendency for cancer cells to recur.

Investigations into PNI in other cancer forms have revealed its potential as a prognostic tool. For early-stage hepatocellular carcinoma, a low PNI is associated with decreased overall and disease-free survival (30). In laryngeal squamous cell carcinoma, PNI is incorporated into predictive models, along with other inflammatory and immune indicators, to effectively predict postoperative recurrence risk (31). These studies indicate that PNI is not just a nutritional status indicator; it may also reflect the overall immune status of cancer patients, which could be of significant importance for predicting TNBC recurrence. The role of PIV and PNI in predicting TNBC recurrence may be similar to their performance in other cancers, involving complex interactions between the tumor microenvironment, immune status, and genetic background.

This study has certain limitations. As a single-center retrospective study with a limited sample size, it may be subject to selection bias. The data in this study have not been externally validated, and the accuracy and stability of the model need further verification. Future studies can expand the sample size and conduct multicenter randomized controlled clinical trials to further confirm the research conclusions. Future research can also further explore the potential mechanisms of PIV and PNI in predicting TNBC recurrence, providing a more scientific basis for clinical treatment.

## 5 Conclusion

In summary, PIV and PNI can serve as effective indicators for predicting recurrence within 2 years after radical surgery in TNBC patients. The nomogram model constructed based on these two indicators has high clinical application value and can provide clinicians with a more scientific reference to help alert them to patients’ recurrence risks in advance.

## Data Availability

The original contributions presented in the study are included in the article/supplementary material. Further inquiries can be directed to the corresponding author.
